# Provision of the frequency modulation system for the hearing impaired^☆^

**DOI:** 10.1016/j.bjorl.2020.03.004

**Published:** 2020-04-27

**Authors:** Monique Ramos Paschoal Dutra, Maria Ângela Fernandes Ferreira

**Affiliations:** a Universidade Federal do Rio Grande do Norte (UFRN), Programa de Pós-graduação em Saúde Coletiva, Natal, RN, Brazil; b Universidade Federal do Rio Grande do Norte (UFRN), Departamento de Odontologia, Centro de Ciências da Saúde, Natal, RN, Brazil

**Keywords:** Hearing loss, Hearing aids, Unified health system, Information systems, Perda auditiva, Auxiliares de audição, Sistema Único de saúde, Sistemas de informação

## Abstract

**Introduction:**

The personal frequency modulation system is an assisted technology resource used in the accessibility of individuals with hearing impairment, being very important in the school environment. Ordinance number 1274/2013 included this device into the Brazilian unified health system.

**Objective:**

To analyze the distribution the personal frequency modulation system concession in the national territory by the Brazilian unified health system from 2013 to 2017.

**Methods:**

This is an ecological study that uses the 27 federation units and the five geographic regions as analysis units. The population involved students in the age range of 5–17 years who reported severe hearing impairment. The data sources were the ambulatory information system/Brazilian unified health system, the Brazilian institute of geography and statistics and the report no. 58 of the national commission for the incorporation of technologies into Brazilian unified health system, national commission of technologies incorporation of the ministry of health. The spatial analysis was carried out through the percentage of coverage of the frequency modulation system concession per federation unit; the data were then unified in the five geographic regions and descriptive statistical analysis was performed.

**Results:**

Between the first and second years, there was an increase of almost 50% in the quantity dispensed, however, there was a progressive decline in the following years. The average coverage of the frequency modulation system in Brazil was 33.4%. The spatial exploratory analysis showed there is a low and uneven distribution in the territory, as the South and Southeast regions had the highest coverage and the North and Northeast regions the lowest, except for the Rio Grande do Norte unit.

**Conclusion:**

The frequency modulation system concession was below that stipulated by the ministry of health and from 2014 to 2017, the dispensed quantity progressively decreased. There is an unequal distribution in the territory that can be explained by the geographic disposition of hearing health services.

## Resumo

**Introdução:**

O sistema de frequência modulada pessoal é um recurso de tecnologia assistida utilizado na acessibilidade das pessoas com deficiência auditiva, sendo muito importante no ambiente escolar. A portaria n° 1.274/2013 inseriu esse dispositivo no sistema único de saúde.

**Objetivo:**

Analisar a distribuição no território nacional da concessão do sistema de frequência modulada pessoal pelo sistema único de saúde no período de 2013 a 2017.

**Método:**

Trata-se de um estudo ecológico e utilizam como unidade de análise as 27 unidades de federação e as cinco regiões geográficas. A população foram os estudantes na faixa etária de 5 a 17 anos que referiram ter deficiência auditiva com comprometimento severo. As fontes de dados foram o sistema de informações ambulatoriais/sistema único de saúde, instituto brasileiro de geografia e estatística e o relatório n° 58 da comissão nacional de incorporação de tecnologias no sistema único de saúdedo ministério da saúde. Foi realizada a análise espacial, através da porcentagem da cobertura da concessão do sistema frequência modulada por unidade de federação e em seguida, os dados foram unificados nas cinco Regiões geográficas e realizada a análise estatística descritiva.

**Resultados:**

Entre o primeiro e segundo ano foi observado um aumento de quase 50% da quantidade dispensada, porém nos anos seguintes constatamos um declínio progressivo. A média da cobertura do sistema frequência modulada no Brasil foi de 33,4%. Na análise exploratória espacial observamos que existe uma baixa e desigual distribuição no território, as regiões Sul e Sudeste apresentaram as maiores coberturas e as regiões Norte e Nordeste as menores, exceto para a unidade do Rio Grande do Norte.

**Conclusão:**

A concessão do sistema frequência modulada foi abaixo do estipulado pelo ministério da saúde e de 2014 a 2017 o quantitativo dispensado diminuiu progressivamente. Existe uma distribuição desigual no território que pode ser explicado disposição geográfica dos serviços de saúde auditiva.

## Introduction

The personal frequency modulation (FM) system is an assisted technology resource used in the accessibility of individuals with hearing impairment, being very important in the academic environment, since it allows students to better perceive the teacher's voice in the classroom, regardless of distance and environmental noise.^1^

This procedure was added by the ministry of health to the list of procedures, drugs, orthoses, prostheses and special materials (OPM) of the Brazilian unified health system (SUS), through ordinance number 1274, of June 25, 2013. The ordinance determines that prescribing the FM system Kit to the child and/or young individual with hearing loss must follow these criteria: having hearing loss and being a user of an individual sound amplification device (ISAD) and/or cochlear implant (CI); mastery of oral language or being in the development phase of oral language; being enrolled in elementary school or high school; and showing a performance in assessing speech recognition skills in silence. The provision of the device should be carried out after a trained multiprofessional assessment, which includes a speech therapist, a doctor, a social worker and a psychologist, and which must consider the opinion of the teacher who follows the child at school.^2^

The report no. 58 of the national commission for the incorporation of technologies in the Brazilian SUS (CONITEC) made an estimate of the number of devices to be dispensed based on the budgetary impact. Data from the 2010 census made it possible to estimate the prevalence of people with hearing impairment in the category “cannot at all” listen in the population age group between 5 and 17 years old. Then, it projected an annual provision of 9738 units of FM systems from 2013 to 2017 and replacement of 9738 units in 2017 in the Brazilian territory.^3^

Since then, few studies have been carried out to evaluate the implementation of the FM system in SUS, most of which report on the device benefits, through the application of instruments to assess speech perception in noise or the perception of parents and teachers.^4^, ^5^, ^6^

Considering that the FM system is a high cost device, that there is an assistance inequality in the territories of Brazil and its important role in enabling full social inclusion, this study aims to analyze the distribution in the national territory of the frequency modulation system concession by the Brazilian unified health system from 2013 to 2017.

## Methods

This is an ecological study that uses the national territory as its base. The study was carried out with secondary data and the analysis units were comprised of the 27 federation units and the five geographic regions. The reference population consisted of students aged 5–17 years who reported having severe hearing impairment.

To calculate the distribution of the frequency modulation system, the ambulatory information system (SIA/SUS) and the Brazilian institute of geography and statistics (IBGE) were used as the data source.

To calculate the distribution of the frequency modulation system, the ambulatory information system (SIA/SUS) and the Brazilian institute of geography and statistics (IBGE) were used as the data source,^7^ together with the report no. 58 of the national commission for the incorporation of technologies in SUS (CONITEC) of the ministry of health.

The data survey was carried out using the DATASUS platform, through the link “Health Information”, restricting the search to “outpatient procedure by place of care” and having Brazil as the geographic scope, with the analysis unit being discriminated by “Federation unit” and, as the period, “January 2013 to December 2017”. The selected procedure was “personal frequency modulation system”, code 0701030321. The selected period was due to the report no. 58 of the national commission for the incorporation of technologies in SUS (CONITEC) of the ministry of health.

Then, at the IBGE website, in the 2010 demographic census link, we selected by state, age range 5–17 years old having reported hearing impairment and “cannot at all”, to obtain the study sample.

The data were collected and organized using the Tabwin software, made available by the Department of Informatics of the ministry of health. The distribution of the frequency modulation system for SUS users was calculated using the following formula: Percentage of FM system concession coverage = nSFM/nDA × 100, where nSFM is the number of concessions of the FM system and nDA is the number of subjects in the age range of 5–17 years, who reported hearing impairment and “can’t do it at all”.

For the spatial analysis, the digital cartographic database of Brazil was used with the federation units, obtained from IBGE, and the map was created, showing that the intensity of the gray levels is directly related to the magnitude of the percentages, that is, the darker the gray level, the higher the values. The data were then unified in the five geographic regions and a descriptive analysis was performed, with mean, minimum and maximum coverage values.

According to the resolution 466/12 of the national health council, public data without the identification of human beings do not need to be submitted to a research and ethics committee, preserving the right to publish the sources without exposure to risk or damage.

## Results

Based on the initial data, we observed that the 27 federative units received a total of 17,707 devices in the period from 2013 to 2017 for a total of 52,976 students with severe hearing impairment.

**Figure 1:**
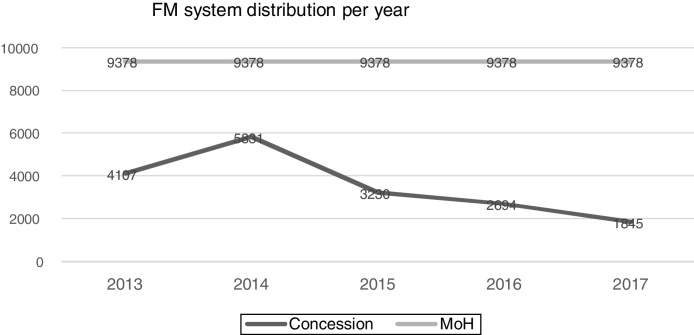
Quantitative distribution of the frequency modulation system per year in Brazil. MoH, Ministry of Health.

Fig. 1 shows the distribution of the frequency modulation system during the studied period and the value recommended by the ministry of health. Between the first and second years, there was an increase of almost 50% in the dispensed amount, but a progressive decline was observed in the following years. The year with the highest provision number was 2014, which still corresponds to approximately 66% of the amount stipulated for the year.

**Figure 2:**
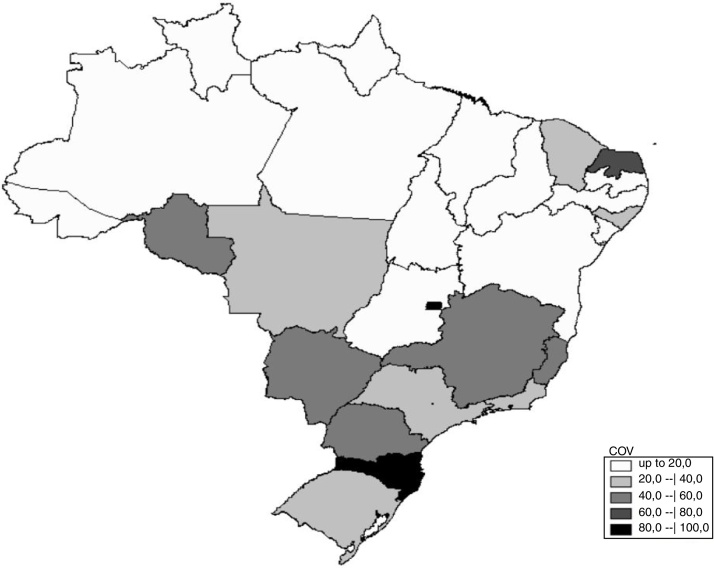
Spatial distribution of the percentage of the frequency modulation system concession per federation unit from 2013 to 2017. COV, coverage.

In the spatial exploratory analysis (Fig. 2) we observed that there is a low and uneven distribution in the territory, with the South and Southeast regions showing the highest coverage and the North and Northeast regions the lowest, except for the Rio Grande do Norte unit (73.7%), which stands out as the third best in Brazil.

Table 1 shows the mean coverage and the states that had the lowest and best percentage per each geographic region of Brazil, which confirms the intra and inter-regional inequality. The mean coverage of the FM system in Brazil was 33.4%, and two federation units, Acre and Roraima, did not show any coverage, while 92.8% of children with severe hearing impairment received the FM system in the state of Santa Catarina.

**Table 1:** Distribution of the FM system coverage by geographic region of Brazil.

Region	Mean (%)	Minimum (%)	Maximum (%)
North	10.3	0 (AC and RR)	45.0 (RO)
Northeast	19.3	6.9 (MA)	73.7 (RN)
Midwest	37.3	18.5 (GO)	86.0 (DF)
Southeast	43.1	36.8 (RJ)	58.1 (MG)
South	52.3	38.3 (RS)	92.7 (SC)

## Discussion

The provision of the frequency modulationsystem represents another accomplishment for hearing health in Brazil. Students using the FM system must be part of the Care Network for Persons with Disabilities, created based on the national plan for the rights of persons with disabilities-living without limit, involving all federative entities and actions of education, social inclusion, accessibility and health care.^8^

The present study shows a peak provision of the FM system in the period 2013–2014, which can be justified by the introduction of the device in the SUS,^2^ whereas before it was acquired only through a court order. There is no record in DATASUS about this quantity, and the teams of the hearing health services had already selected the first student candidates to acquire the system.

However, in the following years, i.e. 2014–2017, a decline was observed, which can be explained by the obstacles reported in the literature. First, in relation to teachers, who have difficulties in understanding the use of technological devices, do not know the adequate environment and distance to communicate with the student with hearing loss and the need to check the device used by that student,^4^, ^9^ which consequently causes a reduction in its use. Moreover, there is the fact that speech therapist instruction is still far from an educational reality for children with hearing loss.^10^ Resistance to adherence to the device by adolescents is another obstacle; a study indicates being ashamed of its use, the breaking of the hearing aid and damage to the device itself often make it impossible to use it.^11^

Finally, family involvement interferes with the consistent use of the FM system: the parents often seem to leave the decision to use the equipment in their children's hands, when they should have a more protagonist role, using different mechanisms to influence the consistent and better quality use.^12^ All of these obstacles may have been faced by the hearing health service teams, causing a decrease in the provision of the FM system by SUS.

Prior to ordinance number 1274/2013, which introduced the personal modulation frequency system procedure, there was decree number 6286, of December 5, 2007, which instituted the health at school program – PSE (*programa saúde na escola*), which aims at the permanent integration and articulation of education and health, improving the quality of life of the Brazilian population.^13^ However, we observe that even after the program updates,^14^ the hearing health actions of the PSE do not address the FM system, which would be extremely important for strengthening the connection of education and health.

In parallel with the introduction of the FM system in SUS, the ministry of education committed to promote the training of Specialized Educational Assistance teachers on the use of technological resources, which favors the academic development of students with hearing loss.^3^ The teachers, if they have adequate training, can be a facilitator for the FM System use by students with hearing impairment.^10^

Thus, the device provision by SUS is not enough for children to enjoy its benefit; it is necessary to develop and improve the joint actions between the areas of Health and Education to encourage students, parents and teachers regarding its effective use, develop policies for monitoring the device, its replacement and repairs, and consequently increase the number of concessions of the FM system in Brazil.

Regarding the distribution of the FM system in the national territory, there is a significant regional inequality, similar to the study of the neonatal hearing screening coverage, which showed a disproportionate interregional and intraregional distribution, with the best coverage being observed in the South and Southeast regions.^15^, ^16^

Such findings can be justified by the professional availability in large urban centers, since these regions have the largest number of speech therapists in the Brazilian territory,^17^ as well as the greater offer of hearing health services, since in 2010 the South and Southeast regions had achieved their total capacity of the functioning hearing networks, while in the northern region this capacity was only 25%.^18^ For the regions with low provision, the hypothesis of the lack of knowledge of the hearing health teams about the device, as well as the lack of training to overcome the adversities that arose, can be raised.

Possibly, this frequency modulation system distribution is also directly related to the location of the cochlear implant centers, as Rio Grande do Norte and the Federal District are pioneering centers of the CI program, which may have provided the two best coverages in Brazil^19^, ^20^ and the fact that the Southeast region concentrates almost 50% of the total of cochlear implant centers, whereas the North region has only one service.^16^

Despite the remarkable evolution of speech therapy assistance in SUS in recent years, there is still a poor distribution of this assistance and speech therapists among the regions and their numbers are still lower than the ones that meet the population's need.^21^ From 2004 to 2011, there has been an increase in hearing health services and care actions in Brazil since the implementation of the national policy for hearing health care (PNASA, *política nacional de atenção à saúde suditiva*); however, important regional inequalities persist in the provision of services.^22^ These are studies that corroborate the results found.

This context can be understood based on a reflection about social inequalities that were historically constructed, which interfered with the health policy process in Brazil and caused a deficiency in access and in the inadequate configuration of health actions and services.^23^

It is important to highlight that the information systems showed to be strong allies in speech therapy scientific research, as they are used as a source of secondary data to subsidize the performance of differential diagnosis and the planning of speech therapy actions.^16^, ^24^ However, it has been observed that the Ambulatory Information System has no replacement, repair or FM system user follow-up code, which would be important for evaluation and monitoring.

Despite the importance of ecological studies in health surveillance and public policy evaluation processes, these do not replace other forms of investigation based on population surveys or other methods that use primary data.^21^

## Conclusion

The provision of the frequency modulationsystem by SUS represents a great advance for the rehabilitation of hearing impaired children; however, we found that it was below that recommended by the ministry of health and that it has decreased in recent years. Moreover, there is an unequal distribution in the territory, with the best coverage concentrated in the South and Southeast regions, except for the units of Rio Grande do Norte and the Federal District. This finding can be explained by the geographical layout of hearing health services.

This study allowed us to envision a reality in the care of students with hearing loss in Brazil and provides grounds for a reassessment of public policies and greater efforts in the North and Northeast regions.

## Conflicts of interest

The authors declare no conflicts of interest.
